# Bacterial Pollution in River Waters and Gastrointestinal Diseases

**DOI:** 10.3390/ijerph14050479

**Published:** 2017-05-04

**Authors:** Lilia Rodríguez-Tapia, Jorge A. Morales-Novelo

**Affiliations:** Economic’s Department, Metropolitan Autonomous University, Av. San Pablo No. 180, Colonia Reynosa Tamaulipas, C.P. 02200, CDMX, Mexico; jamn@correo.azc.uam.mx

**Keywords:** ecological models, gastrointestinal diseases, health-disease function, water pollution

## Abstract

Currently, one of Mexico’s most severe environmental problems is the high levels of pollution of many of its rivers. The present article focuses on the relationship between total coliform bacteria levels and the increase of human digestive tract diseases in the highly polluted Atoyac River in the central Mexican states of Puebla and Tlaxcala. Pollution has become a potential health hazard for people living in nearby river communities. Based on data collected from six of the most contaminated riverside municipalities, two environmental models were developed taking into consideration the health of the entire population, not simply that of its individual members. Such models estimate a health-disease function that confirm the link between Atoyac River pollution and the incidence of gastrointestinal diseases. The causal relation between pollution and gastrointestinal disease incentivizes the creation of epidemiological and public health programs aimed at reducing the environmental health impact of the pollution associated with the Atoyac River. The results presented here are the first of their kind of this river and will serve as basis for future research exploring other similarly contaminated riparian communities. As the causes of pollution are directly related to the economic development and population growth of the region, further research should be conducted for prevention of diseases, educational programs, water remediation and conservation programs that will have a positive impact on the quality of life of the population presently at risk.

## 1. Introduction

Pollution of rivers, lakes, dams and other bodies of water is one of the most severe environmental problems in Mexico. It originates from discharges of untreated or poorly treated domestic and/or industrial waste. These superficial waters usually contain a wide variety of organic and inorganic pollutants, including suspended solids, solvents, oils, grease, plastics, plasticizers, phenols, heavy metals and pesticides. The poor quality of water bodies is a general problem in developing countries, and to a lesser extent in some regions of western countries [1,2]. Sikder et al. [1] report higher concentrations of dissolved organic carbon, *E. coli* and dissolved metals in some rivers of developing countries than those found in Japanese rivers.

Surface water pollution is a prevalent risk to human health and constitutes a hazard to aquatic animals and plants. The negative impact to a specific region has reached critical conditions in certain areas. It depends on the pollutant, which could be anything from organic waste to heavy metals [3]. Gupta et al. [4] state that the accumulation of heavy metals in aquatic systems has become a problem of great concern throughout the world. Trace quantities of metals present in the environment become part of various food chains and accumulate in plants and animals to levels above permissible levels to both humans and other living organisms. In these studies, the authors show that there is a significant accumulation of heavy metals in the waters, sediments and in muscles of two different fish species from different sites of the Ganges River in Allahabad, India. In addition, research done in the drainage basin of the Aral Sea (Central Asia) registered industrial and agricultural pollutants that are highly dangerous to human health. When copper, arsenic, nitrite, and dichlorodiphenyl-trichloroethane (DDT) accumulate in downstream surface water; they reach values above the reference level in the area [5]. These contaminants already have adverse health effects in people living along the river. Agrawal et al. [6] studied the effects of pesticide pollution in riverine systems and drinking water in India. They found in Delhi, Bhopal, (and other cities), and in some rural areas a significant level of pesticides in fresh water systems and in bottled drinking water samples. The levels of DDT reported by these authors in waters from the Yamuna River in Delhi are among the highest ever reported.

Furthermore, studies carried out in various parts of the world report that potentially pathogenic microbes reach surface water bodies by the discharge of untreated domestic wastewater or by sanitary sewage leaks. For instance, in New Jersey, USA, a study determined the risk of contracting diseases because of the combined sewage discharges to the Lower Passaic River [7]. It was found that the concentrations of pathogens in the Passaic River exceed the basic criteria of water quality for human use. In addition, this water sometimes reaches sewage quality. This study found that the likelihood of contracting gastrointestinal diseases (due to fecal *Streptococcus* and *Enterococcus*) by accidental ingestion of water varied respectively from 0.14 to almost 0.70 for people visiting and doing recreational activities. Also, the exposure scenario for homeless people raises their risk of gastrointestinal disease to 0.88. Moreover, in India, most of its rivers are heavily contaminated by discharges of domestic untreated sewage and by direct discharges from industrial waters [6]. A study from the country concludes that the indiscriminate disposal and release of wastes containing hazardous substances can lead to environmental disturbances. These can be considered as a possible source of stress for the biotic communities that exist in the rivers. The health studies reviewed [6,7]—including the one presented here—indicate that because of the levels of pathogens present in contaminated rivers, contact with water poses and will continue to pose significant risks to human health. In fact, these risks will continue until the discharges are controlled adequately or diminished. In developing countries, microbial contamination of surface water used for drinking, recreation and fishing is a pervasive risk to human health. Additionally, severe contamination by pathogenic microorganisms remains the leading cause of water morbidity and mortality. Nevertheless, it is difficult to establish reliable statistical correlations for each case [3]. In these countries, the inability to identify the source of microbial contamination is not always available. Therefore, it is difficult to apply effective strategies to break the water transmission pathway that cause human diseases. The above difficulties are further compounded due to meteorological conditions (such as storms). These conditions also play an important role in microbial water quality. They suggest that an increase in the frequency of storm events could be associated with climate change and is likely to produce a higher incidence of pathogenic loads [3].

Mexico’s National Water Commission (CONAGUA by its Spanish acronym) is a federal entity in charge of supervising and regulating quality of surface water. This commission reports that the Atoyac River—which is located in central Mexico—is one of the most polluted rivers in the country.

The study (in Spanish) “Environmental and epidemiological evaluation to establish health risk factors due to Atoyac River pollution” [8], reports levels of pollutants that are above the limits established in the NOM-001-ECOL-1996 [9]. García et al. [10] reported the presence of lead and arsenic in the Alto Atoyac region in Tlaxcala, Mexico by evaluating discharges of urban, agricultural and industrial wastewaters reaching the river.

Moreover, the prevalence of pollutants in the Atoyac River, especially in the Alto Atoyac region has severely affected the environment and its inhabitants’ health. The main problems reported by Navarro in an epidemiological study [8] include: eye and throat irritation, colds, gastrointestinal problems, cephalea, dermatological allergies, renal problems, parasitic infections, anemia and leukemia.

The present work aims to assess the health of people living in a sub-region of the Atoyac River that presents a high pollution level. The research focuses on the relationship between total coliform bacteria (TCB) and gastrointestinal diseases by using two ecological models. It aims to determine the role that river pollution has among other factors and its contribution to the incidence of these diseases.

Ecological models are useful for health or epidemiological studies related to the environment. These models look at health issues from a communal standpoint and not only as the sum of the health of its individual members [11]. As such, the relationships found through an ecological model must not be extrapolated to explain individual cases.

In an ecological model, the entire group is studied and the unit of observation consists of geographical areas or different time periods of the same region. Through an ecological model, comparison of groups from different areas allows evaluation of several levels of environmental exposure, which is not possible when a single area or group is studied. The limiting factor in this type of study is the availability of information, which needs to accurately reflect the characteristics of the study areas. Information often comes from administrative or legal records, in this case from the health or environmental authorities. After information has been acquired, it needs to be duly processed to obtain the essential indicators for the region [12].

The two most frequently used ecological models are exploratory and multiple groups [13]. In the next sections, first the exploratory model is described. It consists of a comparison of frequency of illnesses in a geopolitical group during a single period. Afterwards the multiple group model allows to compare the results from different geopolitical groups to evaluate the average level of exposure to contaminants and the rate at which illnesses develop. The approach to relate TCB pollution and rates of incidence involve linear- and multiple regression models.

## 2. Materials and Methods

### 2.1. Study Site

The Atoyac River originates at the Sierra Nevada Mountain in northern Puebla and runs about 10 km over its border into the neighboring state of Tlaxcala, joining the Zahuapan River. As it re-enters in southern Puebla, the river ends at the Manuel Avila Camacho dam, also known locally as “Presa Valsequillo” [14].

**Figure 1:**
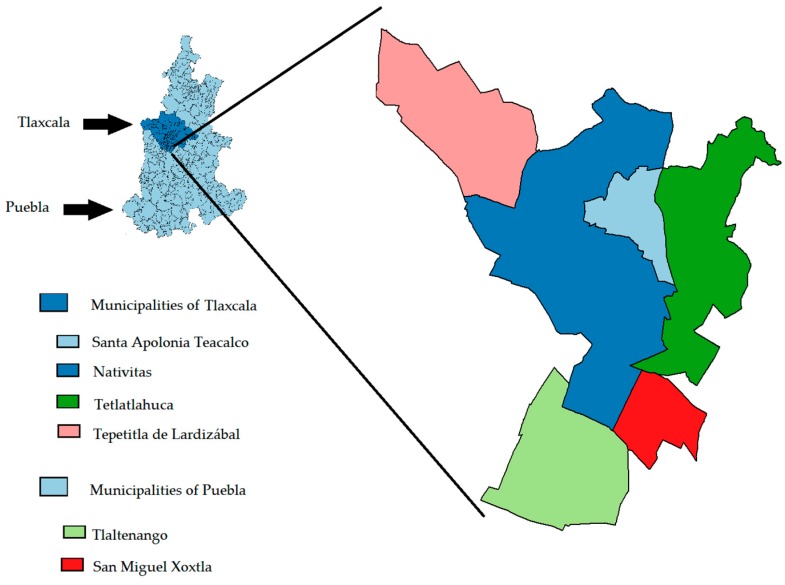
Study region. Municipalities in the states of Puebla and Tlaxcala.

As Atoyac is the main effluent, from now on the Atoyac River will be used to describe the region under study in the Puebla-Tlaxcala area. Along the river, there are six municipalities. Four belong to the state of Tlaxcala: Nativitas, Santa Apolonia, Teacalco; Tepetitla de Lardizábal and Tetlatlahuca. The remaining two belong to the state of Puebla: San Miguel Xoxtla and Tlaltenango (Figure 1).

There is intensive industrial and agricultural activity in the study area. The main industries include textile, petrochemical, plastic, resins, adhesives, food chemicals, paper mills and metallurgy. These activities are not environment-friendly. The river pollution in this zone has its origin in discharges of wastewaters from three industrial sites (Quetzalcóatl, Ixtacuixtla and Huejotzingo), from the Independencia Petrochemical Complex and from over 30 denim-manufacturing plants. Washout from intensive agricultural activity also results in considerable amounts of fertilizers and insecticides reaching the river [8]. Untreated domestic sewage further adds to river pollution, becoming the main source of coliform bacteria.

Despite the high economic importance of the region, the per capita income is lower than that of other urban areas. In general, the socio-economic status of the population is low, which leads to conditions that indirectly impact the environment.

### 2.2. Exploratory Model

The main sources of information for this model were the levels of TCB contamination of the river. These were measured by the Water Commission of the State of Tlaxcala (CEAT) during 2013 [15] and the corresponding patient records from the Nativitas General Hospital, which gives medical coverage to the inhabitants of the study region [16].

### 2.3 Frequency of Gastrointestinal (GI) Diseases in the Study Region

**Figure 2:**
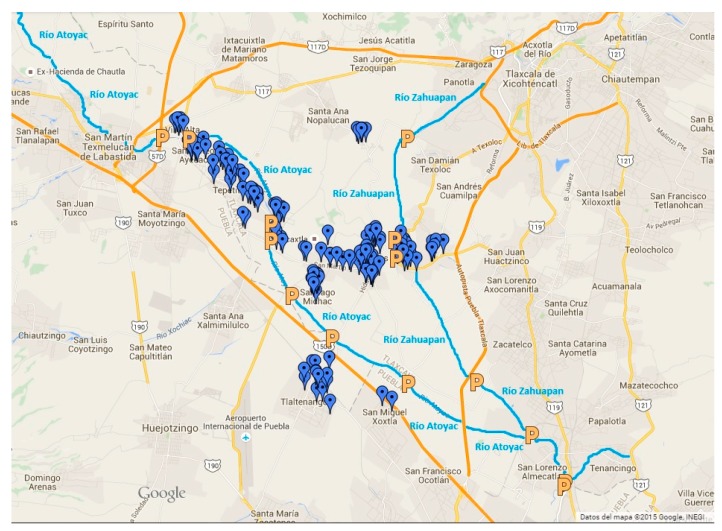
Monitoring sites (P) and pattern of distribution of diseases (in purple) along the Atoyac River. Source: [15,16].

Figure 2 shows monitoring sites along the river, 14 of which are in the study region. These are marked with a letter P in orange color and the sites are in zones with high anthropogenic influence, making them suitable for sampling [17].

Table 1 presents the 2013-monhly average levels of TCB for the different municipalities in the study region. In municipalities with more than one monitoring station, the value listed is the average for all the existing stations. All the values in Table 1 are well above the official Mexican norm NOM-001-ECOL-1996, which sets it at 10^3^ MPN/100 mL (where MPN stands for “most probable number”).

Medical services in the region are provided free of cost by Nativitas General Hospital, with funds from Mexico’s Government. Based on criteria from the attending physicians, out of all cases of gastrointestinal diseases reported in 2013, only those that could be directly related to coliform bacteria contaminating the river were included in the study. There were 123 cases of single-visit outpatients treated and released and 46 who had to be hospitalized for at least 24-h (Table 2).

The 169 cases are marked with purple symbols in Figure 2, which shows the pattern of distribution of diseases, which is clearly distributed along the Atoyac and Zahuapan rivers [17]. The distance between the river and the occurrence of disease is 1.5–3.0 km. This preliminary approach suggests a causal relationship between water contamination and gastrointestinal disease.

Table 2 presents the statistical data on gastrointestinal diseases during 2013 in the six municipalities under study. It is important to note that all the gastrointestinal diseases (GI) reported by the hospital were scrutinized to ensure that cases included in the study were only those confirmed to be from TCB infections.

## 3. Results

The GI incidence rates per municipality are presented in Table 3. The rate of incidence is estimated by dividing the total number of cases by the number of population at risk. A high variability is seen among the different sites.

It is interesting to compare the incidence of Gastrointestinal Disease Rates per Municipality with their annual average levels of TCB, where all the towns are well above the official Mexican norm NOM-001-ECOL-1996.

### 3.1. Simple Lineal Regression Model

The first approach to relate TCB pollution and rates of incidence was a simple lineal regression model using Equation (1):

(1) $$H=b_{0}+b_{1}A$$

where *H* represents the rate of incidence of disease by municipality; b_0_ is the index of GI diseases not related to TCB; *b*_1_ is a factor to relate the rate of disease and exposure and *A* is the level of water contamination.

In other words, the equation simply states that changes of level pollution are directly related to changes of the rate of incidence of TCB- related GI diseases. Leaving out the index of non-TCB diseases, differential Equation (2) is obtained:

(2) $$dH=b_{1}dA$$

stating that rising the level of contamination in one unit the rate of incidence would change in *b*_1_ units [12].

#### 3.1.1. Dispersion of Data, Correlation Coefficient and Linear Regression

**Figure 3:**
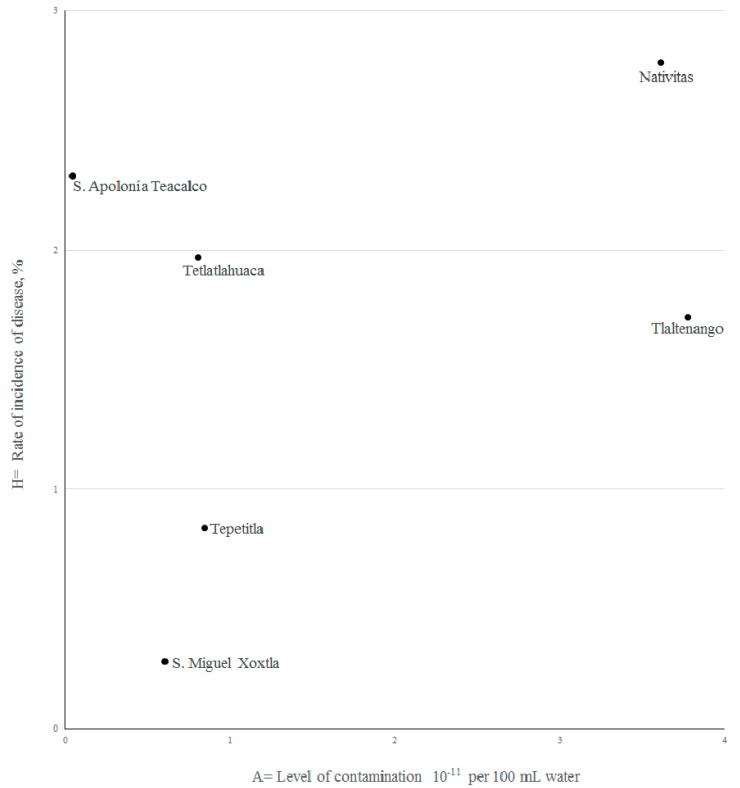
Data scatter plot rate of disease vs. pollution.

Figure 3 shows the scatter of data and the corresponding correlation coefficient, giving a graphical representation of the contamination-disease relationship:

Wide dispersion is seen in this figure, with Nativitas and Tlaltenango with pollution levels that are way over those allowed by the Mexican norm. As there is a limited number of cases, the A-H correlation coefficient is R = 0.41 (R^2^ = 0.168).

#### 3.1.2. Linear Regression

**Figure 4:**
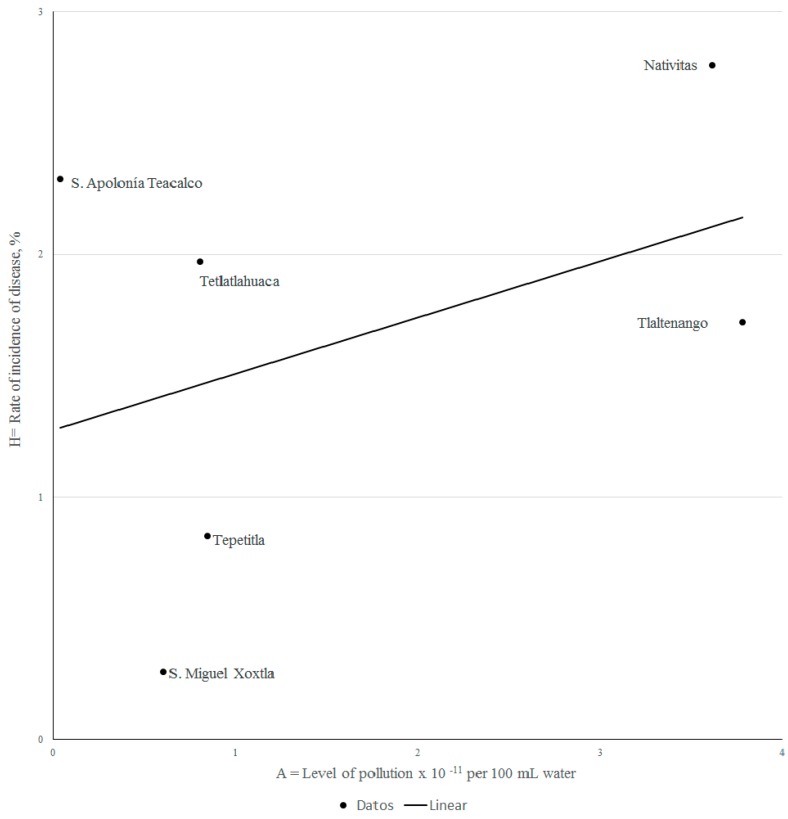
Best-fit linear regression plot of disease vs. pollution.

It is obtained by the least-squares method per Equation (3):

(3) $$H=A\left(2.34\times 10^{-12}\right)+1.269$$

using R^2^ = 0.16 the equation states that the estimated values of *H* reflect only a 16% of the real cases. From the scatter plot a linear regression line can be drawn (Figure 4). Although weak, a positive slope confirms a direct relationship between the measured variables, at least from a qualitative standpoint. Regression test result: Shapiro-Wilk 0.6, Breusch-Pagan 0.48, the pollution regresor test is statistically non-zero with 77% level of confidence with confidence intervals (1.11 × 10^−5^ and 4.76 × 10^−12^).

### 3.2. Multiple Regression Analysis

**Figure 5:**
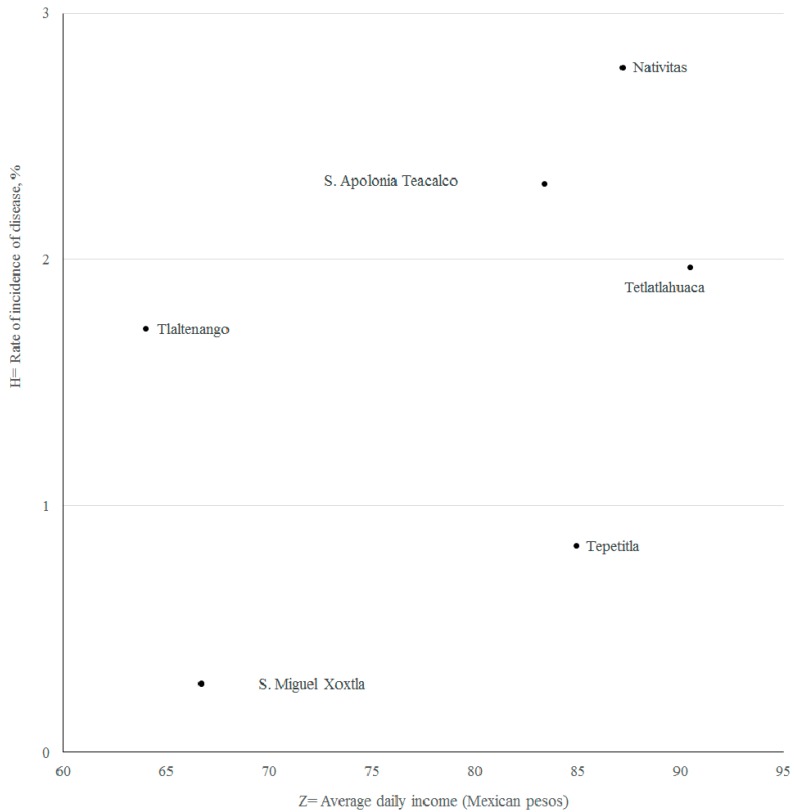
Data scatter plot rates of disease vs. average daily income.

The limited number of data presents a challenge to explain the dependent variable. However, in addition to the information already presented, the patient’s socio-economical information could be obtained, allowing to include a second variable, Equation (4):

(4) $$H=b_{0}+b_{1}A+b_{2}Z$$

in this equation, *Z* is the average daily income of patients per municipality. The scatter plots and correlation coefficients are presented in Figure 5. Adding this new parameter, the new R = 0.5 (R^2^ = 0.58) represents a better fit for the proposed model.

#### Multiple Regression Calculation

The linear regression calculation is now given by Equation (5):

(5) $$H=\left(3.35\times 10^{-12}\right)A+0.055Z-3.3$$

the value of the regresor representing the level of pollution increases from 2.34 to 3.35, but it is still positive and in the same order of magnitude. Regression test result: Shapiro-Wilk 0.88, Breusch-Pagan 0.58, VIF pollution 1.08 and VIF average daily income 1.08, the pollution regresor test report that it is statistically non-zero with 77% level of confidence and gives confidence intervals (2.46 × 10 ^−14^, 6.67 × 10^−12^).

## 4. Discussion

Taking into consideration that in both models, the regresor values are positive and of the same order of magnitude, it could be argued that there is a direct relationship between the incidence of gastrointestinal diseases and the level of river water contamination by TCB.

To show that there is a relationship between contamination and disease, a simulation can be performed using the simple lineal model. It should be remembered that the pollution regresor is in the order of 10^−12^, the high levels of pollution (in MPN/100 mL) range between 10^9^ and 10^11^. Equation (6) can now be used to estimate how variations of the level of pollution influence the incidence of disease:

(6) $$dH=\left(2.34\times 10^{-12}\right)dA$$

focusing on the highly polluted Nativitas site, Table 4 gives the monthly levels of pollution during 2013.

The lowest levels of TCB pollution are found in the July-August months, but in September the level rises from 10^6^ to 10^12^, one million times higher. Per Equation (6) the difference in the disease indicator index is dH ≥ (2.34 × 10^−12^ × 2 × 10^12^) = 4.68. In other words, the percent rise of disease from August to December is at least 4.68%. Nativitas is the community with the largest economic activity in the region, the main activity is manufacturing. This town faces—what is a constant in the region—a lack of environmental regulations so it registers serious pollution problems, highlighting contamination of the river Atoyac. This results suggests that the causes of pollution are directly related to the economic development and population growth, with adverse impacts on the health of the population.

Specific geographical areas are the base units of ecological models, allowing to evaluate the levels of contaminants from a local or regional perspective and to correlate them to differences in health risks. Data collected for ecological models differ in each area depending on the economic or domestic activities, how domestic or industrial waste and other pollutants are disposed and the presence of untreated sewage or sanitary sewer outfalls. Well-designed and executed epidemiological studies are needed for some contaminants for which information on exposure-response relationships is missing or is insufficiently clear.

To-date, there are no data for other diseases that would help to compare the results reported. Nevertheless, the ecological models presented in this study already show a weak but positive causal relationship between some gastrointestinal diseases and Atoyac River contamination by TCB as the main indicator of bacterial pollution of water. Future research should include a larger number of subjects and other socio-economical or climatological factors that might also have an impact on the etiology of gastrointestinal diseases. Possible future increases in the frequency of storm events associated to climate changes are likely to result in a greater incidence of pathogen loads into surface waters [3]. Such studies are significant for governmental programs aimed at helping the inhabitants of contaminated areas. It should also be of help for other epidemiological and animal experiments to clearly establish the mechanisms that lead to increased morbidity in contaminated rivers. As the causes of pollution are directly related to the economic development and population growth of the region, further research should be conducted for prevention of diseases, educational programs, water remediation and conservation programs that will have a positive impact on the quality of life of the population presently at risk.

## 5. Conclusions

Specific geographical areas are the base units of ecological models, allowing to evaluate the levels of contaminants from a local or regional perspective and to correlate them to differences in health risks. Data collected for ecological models differ in each area depending on the economic or domestic activities, how domestic or industrial waste and other pollutants are disposed and the presence of untreated sewage or sanitary sewer outfalls. Well-designed and executed epidemiological studies are needed for some contaminants for which information on exposure-response relationships is missing or is insufficiently clear.

To date, there are no data for other diseases that would help to compare the results here reported, but the ecological models presented in this study already show a weak but positive causal relationship between some gastrointestinal diseases and Atoyac River contamination by TCB, the main indicator of bacterial pollution of water. Future research should include a larger number of subjects and other socio-economical or climatological factors that might also have an impact on the etiology of gastrointestinal diseases. Possible future increases in the frequency of storm events associated to climate changes are likely to result in a greater incidence of pathogen loads into surface waters [3]. Such studies are significant for governmental programs aimed at helping the inhabitants of contaminated areas and should also be of help for other epidemiological and animal experiments to clearly establish the mechanisms that lead to increased morbidity in contaminated rivers. As the causes of pollution are directly related to the economic development and population growth of the region, further research should be conducted for prevention of diseases, educational programs, water remediation and conservation programs that will have a positive impact on the quality of life of the population presently at risk. For instance, a public health program named Popular Insurance expend a lot of money offering free services for poor people for GI diseases. Government will save resources and increases human behalf if apply environmental program regulating the high level of pollution produced by untreated wastewater charges along Atoyac River. The government savings are increased if we include others illness caused by Atoyac River pollution.

## Figures and Tables

**Table 1:** 2013 monthly average levels of total coliform bacteria in six municipalities along Atoyac river.

Municipality	Total Coliform Bacteria, (MPN/100 mL × 10^−10^)
Natívitas	36.1
San Miguel Xoxtla	6.06
SantaApolonia Teacalco	0.428
Tepetitla	8.46
Tetlatlahuca	8.08
Tlaltenango	37.8

Source: [15].

**Table 2:** Number of gastrointestinal disease cases reported during 2013 in the study region.

Municipality	Outpatients	Inpatients	Total
Natívitas	65	14	79
San Miguel Xoxtla	2	1	3
Santa Apolonia Teacalco	8	3	11
Tepetitla	26	15	41
Tetlatlahuca	19	8	27
Tlaltenango	3	5	8
Total cases	123	46	169

Source: [16].

**Table 3:** Incidence of gastrointestinal disease rates per municipality.

Municipality	Total Cases	Potential Cases	Rate of Incidence
			(%)
Natívitas	79	2,841.42	2.78
San Miguel Xoxtla	3	1072.51	0.28
Santa Apolonia Teacalco	11	475.33	2.31
Tepetitla	41	4904.27	0.84
Tetlatlahuca	27	1386.37	1.95
Tlaltenango	8	463.99	1.72
Total region	169	11,143.89	1.52

Source: [16].

**Table 4:** Monthly Level of Pollution in the Nativitas Site (2013).

Month	Level of Pollution
	(MPN/100 mL) × 10^−10^
January	0.021
February	0.450
March	0.430
April	1.2
May	2.1
June	0.94
July	0.0002
August	0.0002
September	220
October	10.3
November	1.8
December	0.067

Source: [15].
